# Supplementary material for: ZmNLR-7-Mediated Synergistic Regulation of ROS, Hormonal Signaling, and Defense Gene Networks Drives Maize Immunity to Southern Corn Leaf Blight
Source: Curr Issues Mol Biol. 2025 Jul 21;47(7):573. doi: 10.3390/cimb47070573 (PMC12293289; doi:10.3390/cimb47070573)
Supplement: Supplementary file 1 [file cimb-47-00573-s001.zip › cimb-3730360-supplementary.pdf]

Supplementary information

Additional file 1: Table S1 Maize EMS mutant plant , (* represents a STOP CODON).

GeneID	Loc	Ref	Mut	Codon_Change	AA_Change	Effect	Mut_Sample
ZmNLR-7-1	93688474	C	T	Caa/Taa	Q/*	STOP_GAINED	EMS4-097d06
ZmNLR-7-2	93694948	C	T	Caa/Taa	Q/*	STOP_GAINED	EMS4-097d0c

Additional file 2: Table S2 Differential expression genes in ET plant hormone signaling pathways

Gene ID	FC	Regulate	KO name
Zm00001eb354640	0.548	down	metK
Zm00001eb055950	0.258	down	ACS
Zm00001eb073290	0.684	down	ACS1_2_6
Zm00001eb089800	0.435	down	ACO, acnA
Zm00001eb234010	0.453	down	ACO, acnA
Zm00001eb191130	2.843	up	ETR, ERS
Zm00001eb191130	2.255	up	ETR, ERS
Zm00001eb216810	1.841	up	ETR, ERS
Zm00001eb119690	0.412	down	EIN2
Zm00001eb244830	0.345	down	EIN3
Zm00001eb331080	0.736	down	EIN3
Zm00001eb307550	0.644	down	ERF1

Additional file 3: Table S3 Differential expression genes in SA plant hormone signaling pathways

Gene ID	FC	Regulate	KO name
Zm00001eb090980	0.918	down	EDS1
Zm00001eb399620	0.369	down	NPR1
Zm00001eb147220	0.605	down	TGA
Zm00001eb197040	0.507	down	TGA
Zm00001eb236380	0.522	down	TGA
Zm00001eb280500	0.274	down	TGA
Zm00001eb352420	0.263	down	TGA
Zm00001eb257300	0.161	down	PR1
Zm00001eb341580	0.185	down	PR1
Zm00001eb341590	0.169	down	PR1

Additional file 4: Table S4 Differential expression genes in JA plant hormone signaling pathways

Gene ID	FC	Significant difference	KO name
Zm00001eb005920	1.184	No	LOX2S
Zm00001eb134020	1.063	No	LOX2S
Zm00001eb058670	1.040	No	AOS
Zm00001eb205210	0	No	AOS
Zm00001eb338800	0.872	No	GH3;JAR1_4_6
Zm00001eb009620	1.091	No	JAZ
Zm00001eb019470	1.295	No	JAZ

Zm00001eb099240	1.039	No	JAZ
Zm00001eb168150	1.091	No	JAZ
Zm00001eb223590	1.33	No	JAZ
Zm00001eb074320	0	No	MYC2
Zm00001eb119000	1.105	No	MYC2
Zm00001eb339360	1.034	No	MYC2
Zm00001eb390330	1.059	No	MYC2

Additional file 5: Table S5 Primer sequences used in the qRT-PCR analysis of Maize

Gene Name	Forward primers（5′→3′）	Reverse primers（5′→3′）
Zm00001eb310440	ACGTCTGTGGCGTTGTTCGT	TTCGGTAGCACGGCGGATTC
Zm00001eb137930	CGGCATGGAGTTCGGCTTCT	CATGTCGCGCATCACCTCCT
Zm00001eb042870	CTCGTCGTGGACCTGCTTGG	AGGCCTTCGCCACTTGTTCC
Zm00001eb230410	CGACGACCTCTTCGACGCAA	GCCGTCGATGAACCTGCGTA
Zm00001eb419870	CGAACGGTGGAGGACGAGTG	CGTTGCAGGCGATGTCGTTG
Zm00001eb341580	GCCGTGCACCCTTCCTACTC	CTGCAGCTTCGTGCTCCAGA
Zm00001eb244830	GCCAAGGAGACGGAGACGTG	CTCTTGAGGCGTCGGCTGAG
Zm00001eb350770	AGGTCATGGCGACGCGAAAT	GGTACACGCCTCGGCTATGG
Zm00001eb122500	ACCGGATCGCCACCATGTTC	GTAGCCCTCGGACCACATGC
Zm00001eb401290	ATAGATTGGCACGGCCGCAA	CACTGGCTCTGCTGCTCCAC
ZmActin	GGGATTGCCGATCGTATGAG	GAGCCACCGATCCAGACACT
ZmUbi	GCAGTGCTGCAGTTCTACAAG	GCAGTAGTGGCGGTCGAAGTG

Additional file 6: Table S6 The nucleotide and amino acid sequences of *ZmNLR-7*.

nucleotide sequences	amino acid sequences
ATGCCGCATGGTCACGCCGCAGCCGTGGTGGACCGGCT	
GCTGCGCAGGCTGGGGTCCGCCGCTTGCCGGCTGGAG	
GTGCCCCCGAATATAGACGGGGACCTAGCGCATGTGTG	
GACAACTCTGGCGAGGTTGCAAGATATGCTGGTTAGCC	
TGGAGATCCGTCCGGAGCTGCAAGAATGGATGGGGGAT	
ATCAAGCAGGTCGCGTACGATGTTGAGGATCTGGTCGA	MPHGHAAAVVDRLLRRLGSAACRLEVPPNIDGDLA
TGAATTGGAAGACCACAATAGCATGGAATCTCAGATGA	HVWTTLARLQDMLVSLEIRPELQEWMGDIKQVAYD
GCGGCTGCGTTGCAGTTGGAGAGGAAACAAGATGGTG	VEDLVDELEDHNSMESQMSGCVAVGEETRWCCSCSF
CTGTTCATGTTCGTTTCTCATGCATAGTACAAGAGCGGA	LMHSTRADRMKTIKRRLDFLVKDSVIFSLMQYPFPDV
CAGAATGAAGACAATCAAAAGAAGGTTGGACTTTTTAG	ERFDNEAFDRAAVVGRDNDKAKIKDMILQSNAQKF
TAAAAGATTCTGTCATCTTCAGTTTGATGCAGTACCCTT	SIIPILGLVGLGKTTLARLIFLDQGEGWDFDLRIWISLN
TTCCTGATGTTGAGAGATTTGATAATGAAGCATTTGATA	RKLNIKMIASDIISQCNHREEKLLDVRTDMEIQENFQ
GAGCTGCAGTCGTTGGAAGAGATAATGACAAAGCAAA	LLKRCLQEALHEKHCLIVLDDLSSTDKNQLDELKEM
GATAAAGGACATGATCTTGCAAAGTAATGCACAAAAGT	LKGTNESIKVLVTTSSEITAELLHTIPPYKLCPLSEDDS
TCTCAATCATTCCCATTCTTGGCCTCGTGGGGTTGGGGA	WKIFSQKAFGNCDGDNTDLKKIGKEIVKRCEGIPLLT
AAACAACTCTTGCTAGATTAATTTTCCTTGACCAGGGAG	HSLGLVVQNEVTNVWLAARDEEIWKLERRVATKIEL
AAGGTTGGGATTTTGATCTTCGTATCTGGATATCCTTAAA	FSPLYQIYNDFSSTIKLCFLYLSIFPKGSTIDKEKLIQQW
TAGGAAACTAAACATAAAAATGATTGCTAGTGATATAAT	IALEIIGSKHDSLPPYVNGEMCIQDFLSIYFLQVRDTH
CTCGCAATGTAATCATAGAGAAGAAAAGCTTTTAGATG	SVYGMDNRVVPTTFYIHNFVHEFARHVACDDIIIFDG
TTCGTACGGACATGGAGATCCAGGAAAACTTTCAGTTG	TKMQKGCAK
CTAAAGAGGTGTTTGCAAGAGGCACTCCATGAAAAAC	
ATTGTCTGATTGTCTTGGATGACCTTTCTAGCACAGATA	
AAAACCAGTTGGATGAATTGAAGGAAATGCTGAAGGG	
TACAAATGAGTCTATCAAGGTTTTAGTGACGACCTCCA	
GCGAAATAACTGCAGAACTACTGCACACCATTCCACCG	

TACAAGTTGTGTCCTTTATCTGAAGACGACAGTTGGAA
AATATTTTCTCAAAAGGCTTTTGGGAATTGTGATGGTGA
TAACACTGACCTTAAGAAAATCGGAAAGGAAATAGTA
AAAAGGTGTGAGGGAATACCATTATTGACTCATTCTCTT
GGTTTAGTTGTACAAAATGAAGTCACAAATGTGTGGTT
AGCTGCAAGGGATGAAGAGATATGGAAACTAGAAAGA
AGAGTTGCCACTAAAATAGAATTGTTTTCACCATTATAT
CAAATATACAATGACTTCTCCTCAACTATTAAATTGTGC
TTTCTATATTTATCCATATTTCCTAAAGGGTCTACAATAG
ATAAGGAAAAACTGATCCAACAGTGGATTGCACTCGA
GATTATTGGATCAAAACATGACTCCTTGCCTCCTTATGT
GAATGGAGAGATGTGCATCCAGGACTTTTTGTCAATATA
TTTTCTCCAAGTTCGAGATACACACTCAGTTTATGGAAT
GGATAACAGAGTAGTTCCTACAACATTCTACATTCATAA
CTTTGTCCATGAATTTGCAAGACATGTTGCTTGTGATGA
TATTATAATTTTCGATGGTACCAAAATGCAAAAGGGCTG
TGCAAAA

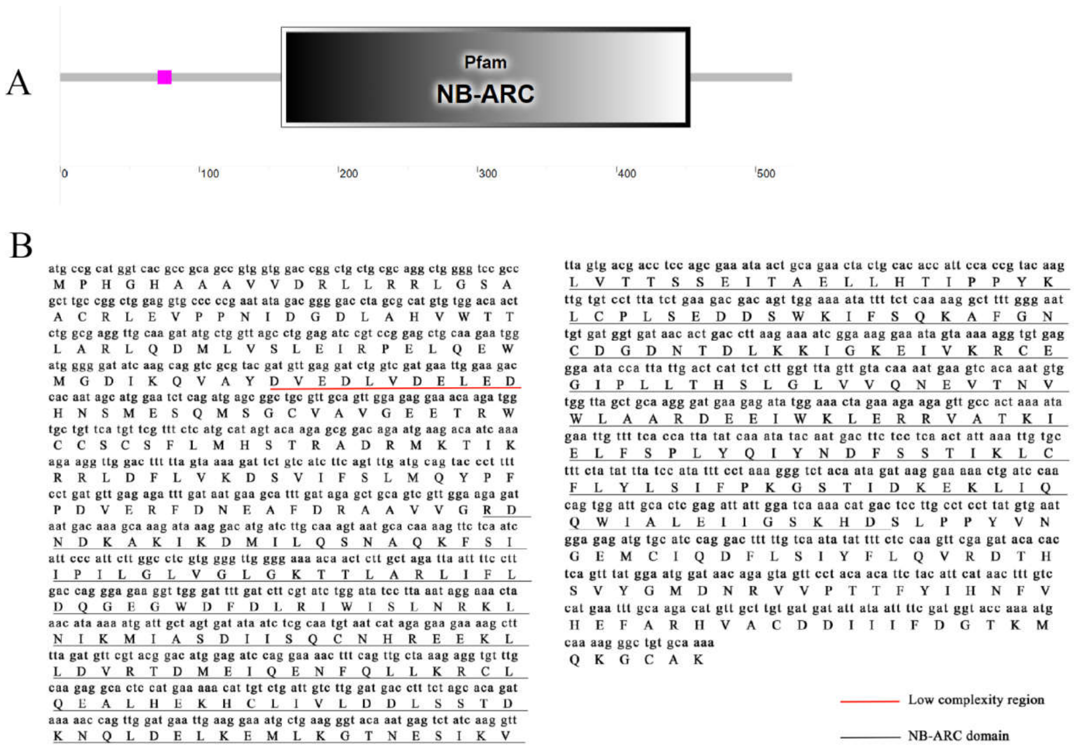

Additional file 7:Figure. S1 (A,B) Molecular character of *ZmNLR-7* gene.

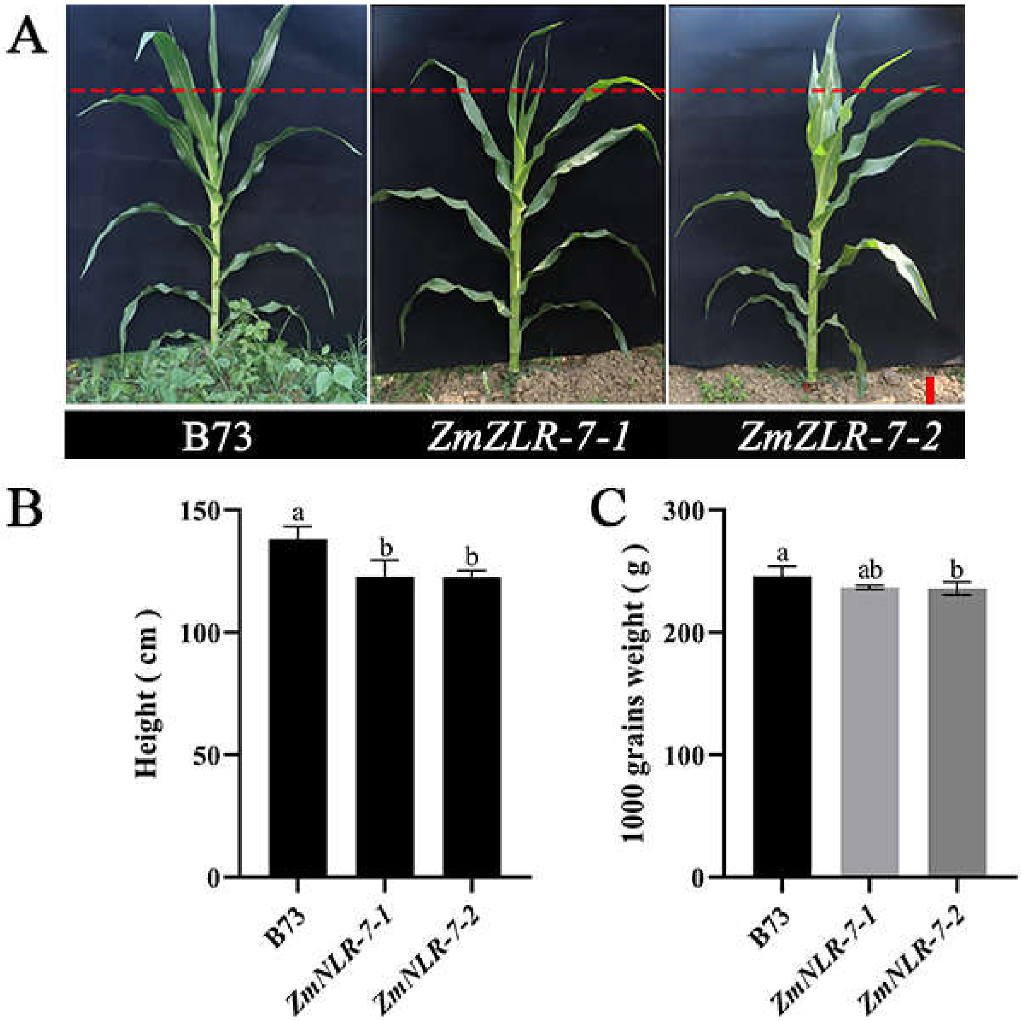

Additional file 8:Figure. S2 Field growth phenotype identification of ZmNLR-7 mutant lines in maize. (**A**)Identification of field growth phenotype;Scale bar: 10 cm; (**B**) Plant height determination in field; (**C**) Weight statistics of thousand grains. Significant differences (P‐value < 0.05) are indicated by different lowercase letters.

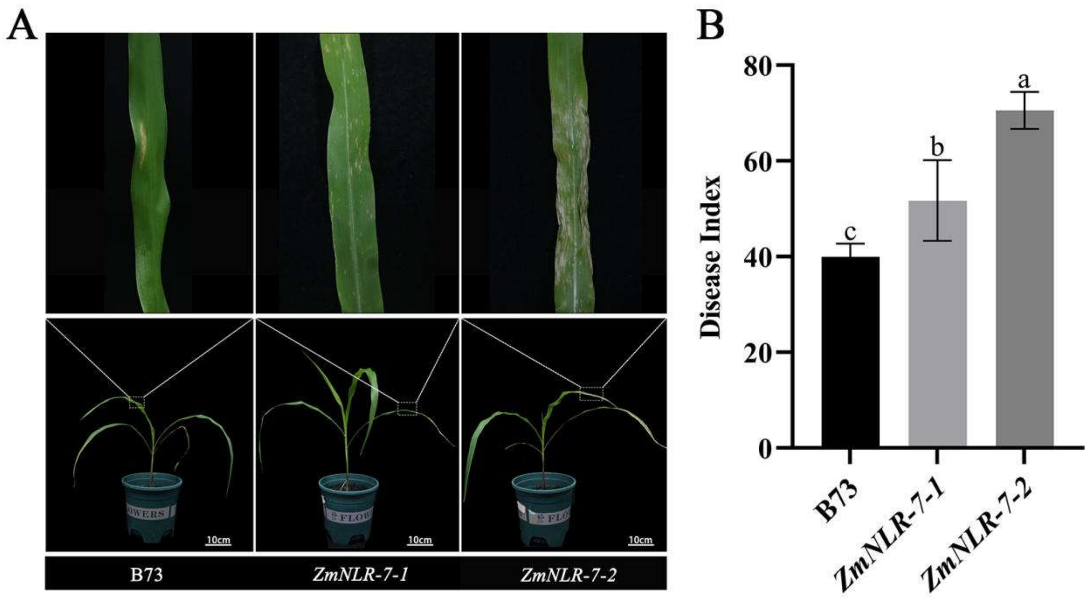

Additional file9:Figure. S3 Analysis of disease resistance function of *ZmNLR-7* mutant lines

(**A**) The resistant phenotypes of different strains on the seventh day after inoculation; (**B**) Disease index of different strains.

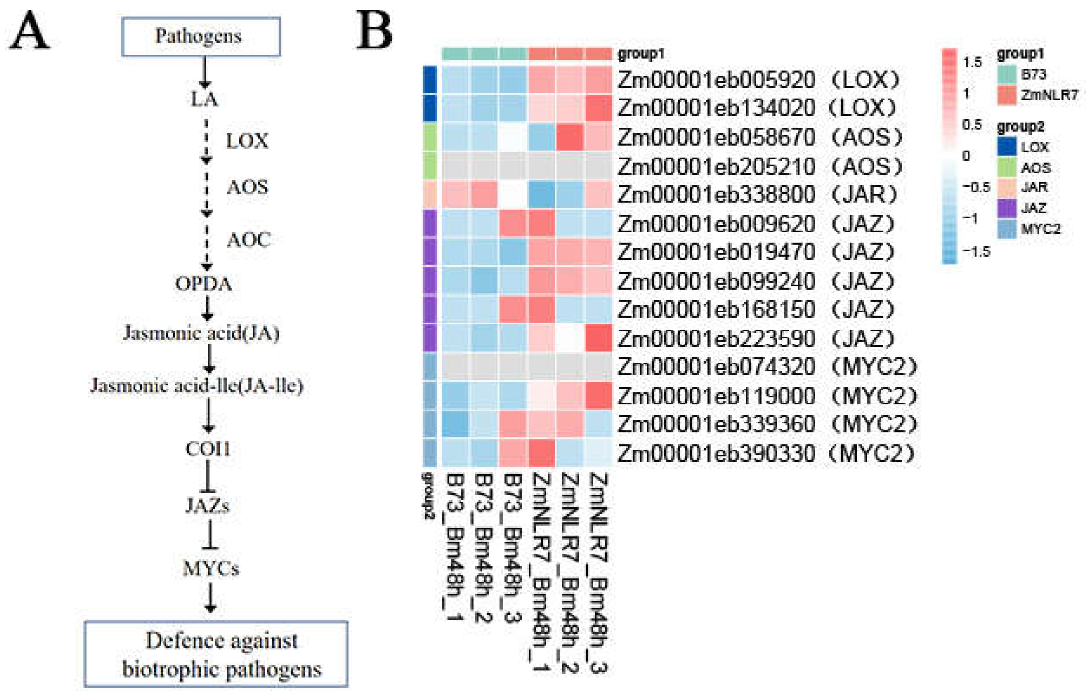

Additional file 10:Figure. S4 The jasmonic acid signaling pathway in ZmNLR-7 and the related gene expression heat map. (**A**) Schematic representation of jasmonic acid signaling pathways; (**B**) Heatmap visualization of jasmonic acid -responsive gene expression levels.

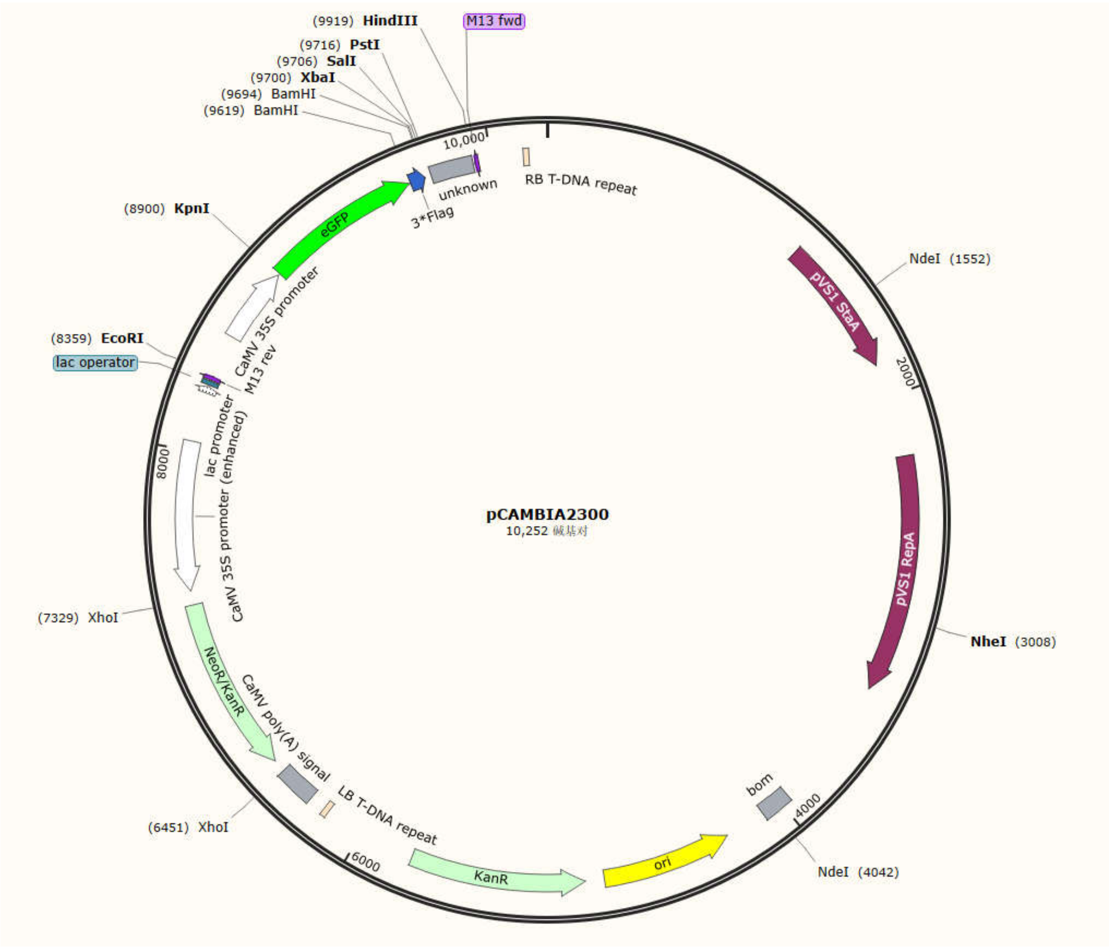

Additional file 11:Figure. S5 The Vector map of *pCAMBIA2300-GFP*.
